# Discrimination and Geographical Origin Prediction of *Cynomorium songaricum* Rupr. from Different Growing Areas in China by an Electronic Tongue

**DOI:** 10.1155/2018/5894082

**Published:** 2018-11-22

**Authors:** Jiaji Ding, Caimei Gu, Linfang Huang, Rui Tan

**Affiliations:** ^1^College of Medcine, Southwest Jiaotong University, Chengdu 610031, China; ^2^Institute of Medicinal Plant Development, Chinese Academy of Medical Sciences & Peking Union Medical College, Beijing 100193, China

## Abstract

*Cynomorium songaricum* Rupr. is a well-known and widespread plant in China. It has very high medicinal values in many aspects. The study aimed at discriminating and predicting *C. songaricum* from major growing areas in China. An electronic tongue was used to analyze *C. songaricum* based on flavor. Discrimination was achieved by principal component analysis and linear discriminant analysis. Moreover, a prediction model was established, and *C. songaricum* was classified by geographical origins with 100% degree of accuracy. Therefore, the identification method presented will be helpful for further study of *C. songaricum*.

## 1. Introduction

**Figure 1:**
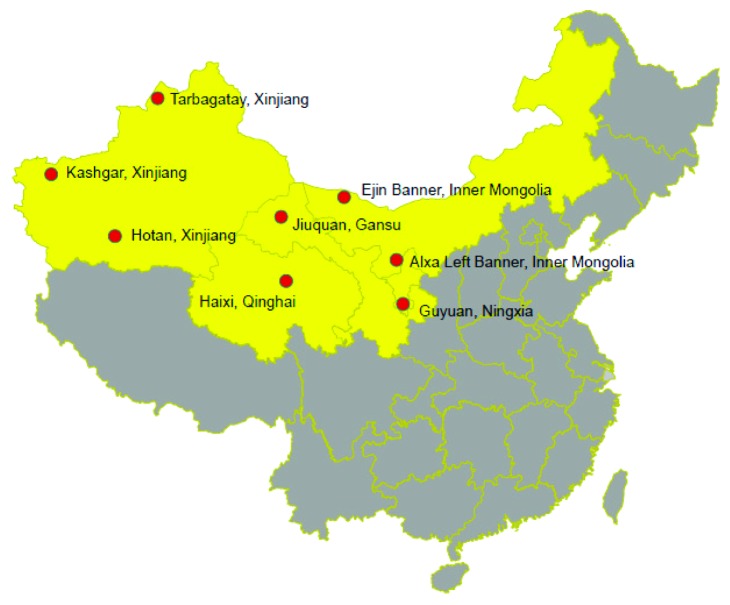
The distribution of *Cynomorium songaricum* Rupr. in China.

*Cynomorium songaricum* Rupr. of the family *Cynomoriaceae* is a desert, holoparasitic perennial plant found in China, Mongolia, Iran, and Afghanistan [1, 2]. In China, *C. songaricum* grows in Xinjiang, Inner Mongolia, Ningxia, Qinghai, and Gansu [3] (Figure 1). *C. songaricum*, called *Suo Yang* in China, is a known food, nutrient, and a tonic herb for improving kidney and immunity function and treating constipation [4, 5]. This plant is one of the most popular herbs in the world and is documented in some famous medicinal works [1]. Various compounds, including flavonoids, organic acids, steroids, saccharides, terpenoids, phloroglucinol adducts, phenylpropanoids, and other types of compounds, have been isolated from *C. songaricum* to date [1, 6]. These chemical compounds exhibit numerous biological activities, including antiapoptosis, antifatigue, antioxidant, antiosteoporotic, antiaging, antidiabetic, anti-HIV protease, anti-HCV protease, and fertility promotion [1, 7–9].

In our previous work, we presented that the chemical constituents of *C. songaricum* from different producing areas vary, thus affecting the quality of the plant [2]. Genuine medicinal herb, which means Daodi yaocai in Chinese, is a unique definition in traditional Chinese medicine. Medicinal herbs growing in a specific place exhibit high quality [10]. Currently, chromatographic herbal fingerprints have become one of the most applied quality control tools for similarity analyses of herbal medicines [11]. However, it costs a relatively long time. Thus, a more convenient way for identification and quality control of herbs is needed.

Electronic tongues are analytical systems formed from an array of electrochemical sensors combined with data-processing tools intended to interpret electrochemical signals. Similar to human receptors, the sensors of an electronic tongue undergo a series of reactions. While the generated reactions differ from one another, the information acquired from each sensor is complementary. Then, the results combined by the sensors generate a unique fingerprint that can reflect the macroscopic characteristics of samples. In biological mechanisms, gustatory signals are transducted by brain nerves in the form of electric signals. Electronic tongue sensors approach flavors similarly, given that electric signals are generated with potentiometric variations. The perception and recognition of taste quality are based on the recognition or building of activated sensory nerve patterns in the brain and the gustation fingerprint of a product. This step is accomplished by the statistical software of the electronic tongue that can translate sensor data into taste patterns [12–19]. In the recent years, electronic tongues have been commonly used to analyze food and beverages, given their advantages of short response time, strong objectiveness, human safety, and repeatability [20]. As for some herbs like *C. songaricum*, they have different tastes and flavors according to different places of origin and are ready to eat. Based on this, the simplicity and convenience of electronic tongues could be used in the analysis of the herbs.

In this work, we first developed a method to discriminate and predict the geographical origin of *C. songaricum* from different growing areas in China by using an electronic tongue. Pattern recognition techniques, including principal component analysis (PCA) and linear discriminant analysis (LDA), were used for data analysis in this research. In addition, this study provided a simple approach for identifying the geographical origins of *C. songaricum*, and the acquired information can be used for evaluating the quality of *C. songaricum* growing in China.

## 2. Materials and Methods

### 2.1. Samples

*C. songaricum* samples were collected from different areas in China (Kashgar in Xinjiang, Tarbagatay in Xinjiang, Jiuquan in Gansu, Guyuan in Ningxia, Hotan in Xinjiang, Haixi in Qinghai, Ejin Banner in Inner Mongolia, and Alxa Left Banner in Inner Mongolia) (Table 1). All of the samples were authenticated by Professor Linfang Huang in the Institute of Medicinal Plant Development, Chinese Academy of Medical Sciences, and Peking Union Medical College, Beijing, China.

### 2.2. Instrument

The electronic tongue system (taste sensing system Astree II, France) consists of a reference electrode and seven liquid sensors (ZZ, JE, BB, CA, GA, HA, and JB) with a cross-selection function, a fully automated sample injector, and a personal computer with a software for sample injection, data acquisition, and chemometric analysis.

### 2.3. Experimental Procedures

Pieces of each sample (10 g) were placed in a beaker, soaked with 200 mL of pure water for 30 min, and then decocted for 30 min. The solution was filtered immediately. The residue was processed according to the abovementioned method twice. Afterward, all filtrates were combined. The obtained solution was placed into the special beaker of the electronic tongue and detected at room temperature.

Each sensor collected data from each sample for 120 s and was cleaned for 10 s. Then, data were recorded by the data acquisition system. All assays were carried out in triplicate.

### 2.4. Pattern Recognition

In this paper, PCA and LDA were used to differentiate *C. songaricum* originating from different places.

PCA is a multivariate statistical method that reduces the dimensionality of data while retaining most of the variation in the data [21]. This approach was created before World War II but became widely used during the “Quantitative Revolution” in the 1960s [22]. New linear combinations of variables are created to accomplish the reduction. The combinations, called principal components, characterize the objects studied and satisfy certain statistical and mathematical conditions. Thus, samples can be displayed by few variables, and assessment of similarities and differences among samples is simplified. Thus, PCA is a suitable method to discriminate different samples and is extensively applied in food and drug analysis.

LDA is another commonly used technique for data discrimination and dimensionality reduction. LDA is strongly linked to regression analysis and analysis of variance (ANOVA), which also aims at expressing one dependent variable as a combination of other measurements or features [23]. The method maximizes the ratio of the between-class distance to the within-class distance to guarantee maximum discrimination. LDA has been used in numerous applications, such as image retrieval, microarray data classification, face recognition, and food and beverage discrimination [24–27].

## 3. Results and Discussion

### 3.1. Radar Map

**Figure 2:**
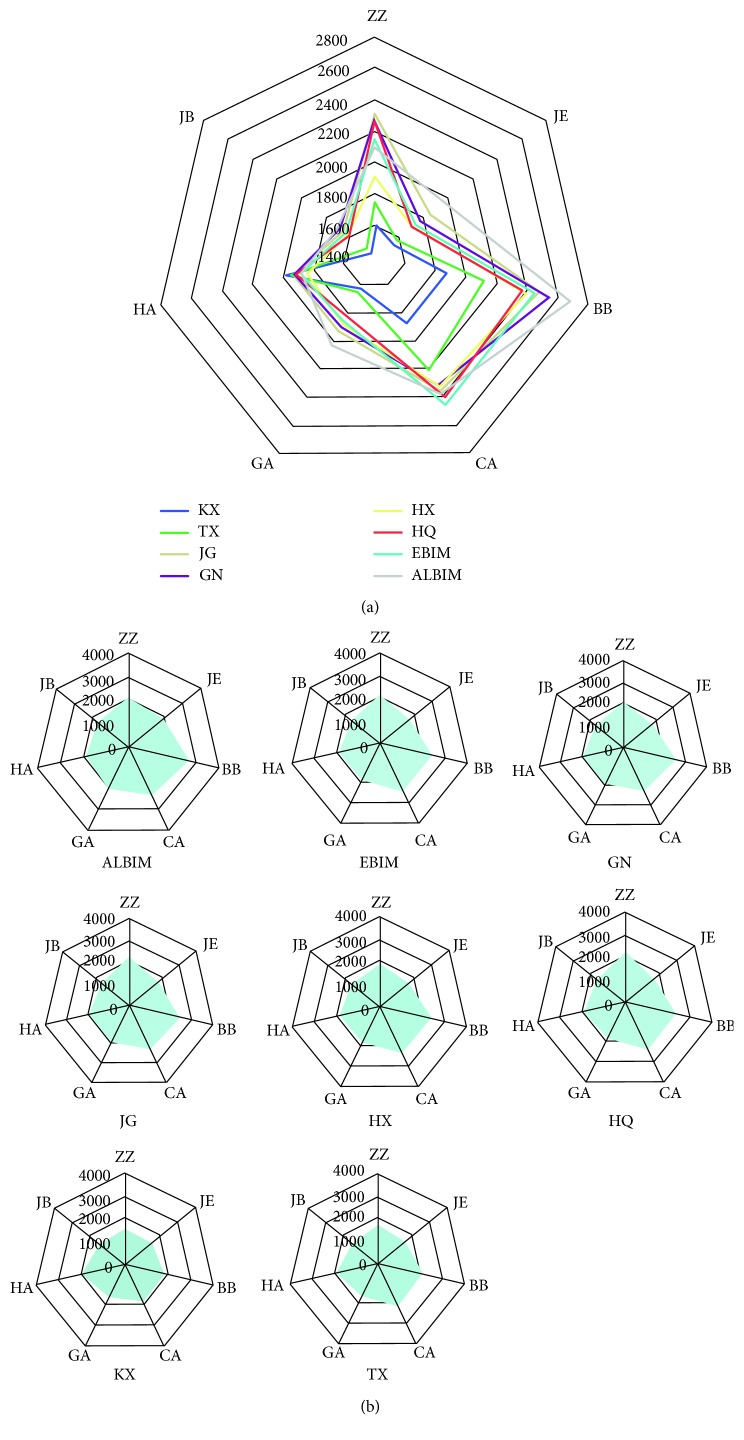
The radar map of *C. songaricum* from different producing areas in China.

Figure 2 shows the radar map of samples from different places in China. The sensor response of the electronic tongue varied with the change in geographical origins. Evidently, sensors BB, CA, and ZZ show strong signals to the samples. In particular, sensor BB exhibits the strongest response to the samples.  Figure 2(a) shows the distinction among all samples from different places clearly. Signals from different samples show a considerable difference.  Figure 2(b) illustrates the signals of every sample separately. Samples KX, TX, and HX are different from others, with the signals of sensor ZZ of these samples not exceeding 1000. Shapes of the radar maps of other samples are similar.

### 3.2. Principal Component Analysis

**Figure 3:**
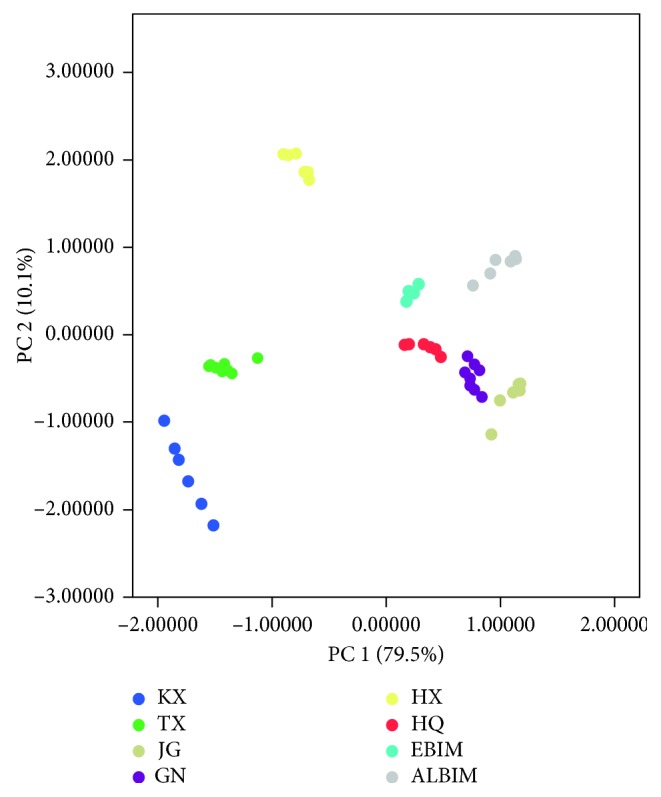
Discrimination of PCA plots for different *C. songaricum* samples.

The first discrimination model was established using PCA to visualize the different *C. songaricum* groups where possible. The accumulated explained variance was 89.6%, which was distributed in 79.5% (PC1) and 10.1% (PC2).  Figure 3 shows the results of PCA score plot, and several trends are observed. Eight types of *C. songaricum* samples can be classified in general. Moreover, *C. songaricum* samples from Xinjiang are discriminated clearly between samples from other provinces. Similar samples appear in the same location of the graph. Thus, *C. songaricum* samples from Gansu, Ningxia, Qinghai, and Inner Mongolia are similar. The chemical constituents of these samples may be similar as well.

### 3.3. Linear Discriminant Analysis

**Figure 4:**
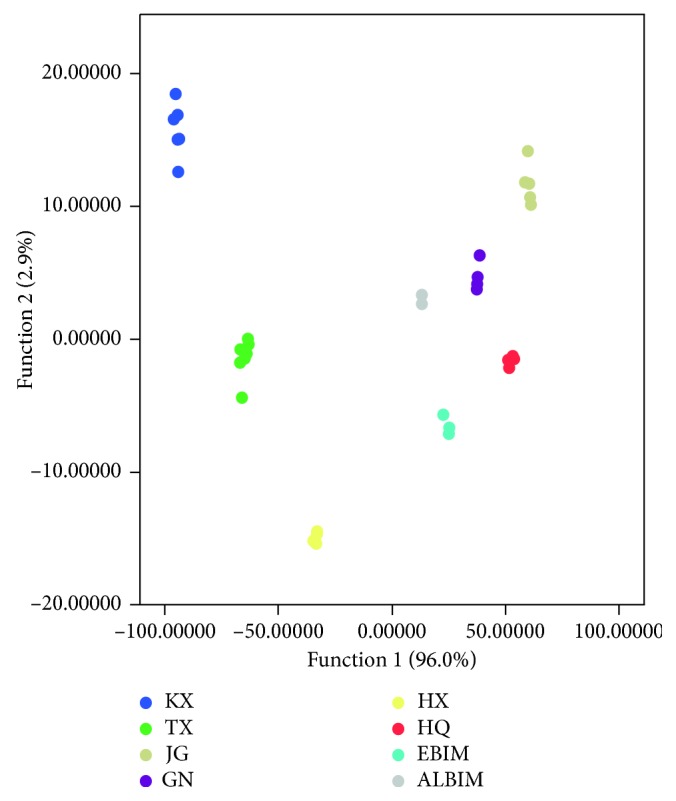
Discrimination of LDA plots for different *C. songaricum* samples.

Figure 4 shows the dispersion of *C. songaricum* samples by the LDA model. Compared with the PCA model, the LDA model shows a clearer discrimination among the eight types of *C. songaricum*. The explained variances by each discriminant function (DF) were 96.0% (DF1) and 2.9% (DF2). Each group of *C. songaricum* samples can be distinctly classified with others. As a result, the LDA model is a superior method to discriminate *C. songaricum* from different growing areas in China.

Given the good discrimination feature of the model, we used the LDA model to predict the geographical origin of unknown *C. songaricum* samples. As shown in Table 2, the prediction model can classify *C. songaricum* by geographical origins with 100% degree of accuracy.

## 4. Conclusions

We applied an electronic tongue to classify and predict *C. songaricum* samples from different places of origin. PCA and LDA were used for discrimination. The LDA model shows a clearer discrimination than the PCA model. The eight groups of *C. songaricum* samples can be classified precisely using the LDA model. On this basis, we used the LDA model to predict the geographical origin of several unknown *C. songaricum* samples. A prediction model with 100% degree of accuracy was achieved. We present for the first time a method for the discrimination and geographical origin prediction of *C. songaricum* from different growing areas in China according to their flavor by an electronic tongue. The operations of data acquisition and processing are simpler and more convenient than the traditional chemical methods. The acquired information can be used for evaluating the quality of *C. songaricum* growing in China according to our previous work. Moreover, the identification and quality analysis method presented by us will be helpful for further study of *C. songaricum*. Further efforts should be focused on investigating the connection between the flavor and chemical constituents of *C. songaricum* samples and correlating electronic tongue signals with human perceptions of taste.

## Figures and Tables

**Table 1:** Sample list of *Cynomorium songaricum* Rupr.

Sample	Place of origin
Discrimination	
KX-1	Kashgar, Xinjiang
KX-2	Kashgar, Xinjiang
TX-1	Tarbagatay, Xinjiang
TX-2	Tarbagatay, Xinjiang
TX-3	Tarbagatay, Xinjiang
JG-1	Jiuquan, Gansu
JG-2	Jiuquan, Gansu
GN-1	Guyuan, Ningxia
GN-2	Guyuan, Ningxia
GN-3	Guyuan, Ningxia
HX-1	Hotan, Xinjiang
HX-2	Hotan, Xinjiang
HX-3	Hotan, Xinjiang
HQ-1	Haixi, Qinghai
HQ-2	Haixi, Qinghai
HQ-3	Haixi, Qinghai
EBIM-1	Ejin Banner, Inner Mongolia
EBIM-2	Ejin Banner, Inner Mongolia
EBIM-3	Ejin Banner, Inner Mongolia
ALBIM-1	Alxa Left Banner, Inner Mongolia
ALBIM-2	Alxa Left Banner, Inner Mongolia
Prediction	
KX-3	Kashgar, Xinjiang
KX-4	Kashgar, Xinjiang
TX-4	Tarbagatay, Xinjiang
TX-5	Tarbagatay, Xinjiang
JG-3	Jiuquan, Gansu
JG-4	Jiuquan, Gansu
GN-4	Guyuan, Ningxia
GN-5	Guyuan, Ningxia
HX-4	Hotan, Xinjiang
HX-5	Hotan, Xinjiang
HQ-4	Haixi, Qinghai
HQ-5	Haixi, Qinghai
EBIM-4	Ejin Banner, Inner Mongolia
EBIM-5	Ejin Banner, Inner Mongolia
ALBIM-3	Alxa Left Banner, Inner Mongolia
ALBIM-4	Alxa Left Banner, Inner Mongolia

**Table 2:** Confusion matrix for LDA prediction method of *C. songaricum* samples from different producing areas.

Actual	Predicted							
	KX	TX	JG	GN	HX	HQ	EBIM	ALBIM
KX	6							
TX		6						
JG			6					
GN				6				
HX					6			
HQ						6		
EBIM							6	
ALBIM								6

## Data Availability

The data used to support the findings of this study are available from the corresponding author upon request.
